# Diurnal Differences in Human Muscle Isometric Force In Vivo Are Associated with Differential Phosphorylation of Sarcomeric M-Band Proteins

**DOI:** 10.3390/proteomes8030022

**Published:** 2020-08-26

**Authors:** Zulezwan Ab Malik, Kelly A. Bowden Davies, Elliott C. R. Hall, Jennifer Barrett, Samuel A. Pullinger, Robert M. Erskine, Sam O. Shepherd, Zafar Iqbal, Ben J. Edwards, Jatin G. Burniston

**Affiliations:** 1 Research Institute for Sport & Exercise Sciences, Liverpool John Moores University, Liverpool L3 3AF, UK; zulezwan@fsskj.upsi.edu.my (Z.A.M.); k.bowden.davies@mmu.ac.uk (K.A.B.D.); elliot.hall@mmu.ac.uk (E.C.R.H.); J.Barrett@2014.ljmu.ac.uk (J.B.); Samuel.Pullinger@aspire.qa (S.A.P.); R.M.Erskine@ljmu.ac.uk (R.M.E.); S.Shepherd@ljmu.ac.uk (S.O.S.); b.j.edwards@ljmu.ac.uk (B.J.E.); 2 Institute of Sport, Exercise and Health, University College London, London WC1E 6BT, UK; 3 Crystal Palace Football Club, London SE25 6PU, UK; Zafar.iqbal@cpfc.co.uk; 4 Liverpool Centre for Cardiovascular Science, Liverpool John Moores University, Liverpool L3 3AF, UK

**Keywords:** maximum voluntary isometric contraction, muscle contraction, phosphopeptide, phosphoproteomic, protein processing, post-translational, proteomics, rate-of-force development, sarcomere, time of day

## Abstract

We investigated whether diurnal differences in muscle force output are associated with the post-translational state of muscle proteins. Ten physically active men (mean ± SD; age 26.7 ± 3.7 y) performed experimental sessions in the morning (08:00 h) and evening (17:00 h), which were counterbalanced in order of administration and separated by at least 72 h. Knee extensor maximal voluntary isometric contraction (MVIC) force and peak rate of force development (RFD) were measured, and samples of vastus lateralis were collected immediately after exercise. MVIC force was greater in the evening (mean difference of 67 N, 10.2%; *p* < 0.05). Two-dimensional (2D) gel analysis encompassed 122 proteoforms and discovered 6 significant (*p* < 0.05; false discovery rate [FDR] = 10%) diurnal differences. Phosphopeptide analysis identified 1693 phosphopeptides and detected 140 phosphopeptides from 104 proteins that were more (*p* < 0.05, FDR = 22%) phosphorylated in the morning. Myomesin 2, muscle creatine kinase, and the C-terminus of titin exhibited the most robust (FDR < 10%) diurnal differences. Exercise in the morning, compared to the evening, coincided with a greater phosphorylation of M-band-associated proteins in human muscle. These protein modifications may alter the M-band structure and disrupt force transmission, thus potentially explaining the lower force output in the morning.

## 1. Introduction

The force output of muscle is a key component of athletic performance, and adequate levels of muscle mass and function are critical to health and well-being in the general population [1]. For example, the power output of muscle becomes a key determinant of independence and quality of life in older individuals [2]. The sarcomere is the basic contractile unit of muscle and consists of an ordered arrangement of thick and thin myofilaments in a three-dimensional lattice. Sarcomeres are arranged in series, bounded by Z-disks that connect with the muscle cytoskeleton, and have a central M-band that connects the anti-parallel thick filaments at the center [3]. Myosin heavy-chain and filamentous actin are the main proteins of the thick and thin filaments, respectively. In addition, the myofibrillar proteome consists of numerous other major proteins, e.g., myosin light chains, troponins, and tropomyosins, as well as lesser-known ancillary proteins whose functions remain enigmatic or are only recently coming to light. Despite its highly ordered structure, striated muscle is renowned for its plasticity and can adapt both quantitatively, through hypertrophy or atrophy, and qualitatively by changing the relative proportion of myofibrillar protein isoforms. Remarkably, the maximum force that can be produced by muscle varies throughout the day and is significantly greater in the evening than the morning [4,5]. The difference in maximum isometric force between the morning and evening is typically approximately 10% [6,7]. For context, this magnitude of difference is equivalent to the gain in muscle force achieved after several weeks of resistance exercise training [8].

Time-of-day differences in maximal isometric force appear to be intrinsic to muscle rather than secondary to fluctuations in neuromuscular drive [9] or body temperature [10]. In the last decades, significant progress has been made in understanding the molecular workings of the central (suprachiasmatic nucleus) and peripheral tissue clocks and their roles in biology [11]. At the molecular level, the circadian clock is a well-defined gene regulatory network composed of transcriptional–translational feedback loops involving proteins such as circadian promotor output cycles kaput (CLOCK) and brain and muscle ARNT like-1 (BMAL1; [12]). Early work in human muscle [13] reported a diurnal difference in the expression of clock genes and the muscle linage gene, myogenic factor 6. More detailed analysis of circadian gene expression in animals [14] identified further muscle-specific genes, including myogenic differentiation protein 1 (MyoD), which exhibits a circadian rhythm. The genetic deletion of key components of the molecular clock continues to be a powerful tool for investigating cellular processes that are dependent on circadian regulation. Muscle-specific disruption of the molecular clock results in impairments to glucose metabolism and insulin homeostasis [15] in mice, and exercise at different times of day is associated with different metabolic responses in muscle [16]. The deletion of BMAL1, whether from germline [17] or induced in adult muscle [18], is associated with losses in muscle contractile force, which may be relevant to the diurnal differences in maximum isometric force observed in humans. However, it is not yet clear how circadian oscillations in gene expression affect hour-by-hour differences in the force output of muscle. The majority of myofibrillar proteins have a half-life of approximately 30 days [19], so it is unlikely that changes in gene expression and protein abundance account for diurnal differences in muscle force production. Instead, the post-translational modification of proteins may be responsible. Modifications such as phosphorylation can occur on second or minute timescales and are known to change the functional properties of myofibrillar proteins [20].

Currently, fewer than 12 articles report analyses of human muscle biopsy samples in the context of chronobiology. Moreover, a proteomic analysis of human muscle in the context of diurnal or circadian rhythm has not yet been reported. Maximal contractions evoke acute changes in muscle protein phosphorylation [21], but it is not yet clear whether an interaction exists between exercise and time of day that could contribute to differences in the force output of muscle or the Zeitgeber effect of exercise on muscle function. Therefore, we used proteomic techniques to investigate the post-translational state of muscle proteins immediately after maximum force contractions in either the morning or evening. Two-dimensional gel-electrophoresis (2DGE) is a useful ‘top–down’ proteomic method for the non-targeted analysis of proteoforms. Proteoforms arise through specific combinatorial patterns of post-translational modification and the splice-variation of protein isoforms. The post-translational modification of specific myosin light chain isoforms alters the contractile properties of striated muscle [22] and could contribute to time-of-day differences in maximal isometric force. Myosin light chain isoforms are readily resolved by 2DGE into proteoforms, which include the post-translational modification of specific amino acid residues that can be identified using mass spectrometry [23]. In addition, the selective enrichment of modified proteins or peptides prior to mass spectrometry is a well-established ‘bottom–up’ proteomic approach to studying post-translational modifications. Often, bottom–up methods offer more comprehensive and higher throughput analysis than 2DGE; however, the isolated analysis of site-specific modifications does not provide proteoform-specific information [24]. Nevertheless, bottom–up analyses can be useful in identifying modifications to key protein residues that may be associated with the difference in muscle force output between morning and evening. Phosphorylation is perhaps the most widely studied post-translational modification. Robust methods for the enrichment of phosphopeptides are established [25]; however, currently, fewer than 6 articles report the phosphoproteome analysis of human skeletal muscle. The phosphoproteome of human muscle has been mapped [26], and the acute response to endurance exercise has been reported [27]. Herein, we investigate whether differences also exist in the muscle phosphoproteome after exercise in the morning versus evening. 

## 2. Materials and Methods

Pilot experiments recruited n = 20 males with age (mean ± SD): 26.0 ± 4.4 years; body mass: 75.1 ± 8.2 kg; height: 177.3 ± 6.8 cm; and normative retiring and rising times: 23:36 ± 0:36 h:min and 06:24 ± 0:18 h:min, respectively. Verbal explanation of the experimental procedure was provided to each individual, including the aims of the study, the possible risks associated with participation, and the experimental procedures. Individuals provided written informed consent before participating in the study. The experimental procedures were approved (14/WM/0065) by the National Research Ethics Committee, UK and conformed to the standards set by the Declaration of Helsinki, except for registration in a database.

Participants were selected if they met all the inclusion requirements, including habitual sleeping duration of approximately 8 h per day, no reported sleep problems, did not routinely nap, had at least 2 years experience in strength or weight training, and habitually trained at any time of the day during a typical week. Participants were also required to avoid alcohol 48 h prior to each test. None of the participants had a history of bone fractures or musculoskeletal abnormalities, and none were prescribed pharmacological treatment during the study. Participants were excluded from participating if they had (i) recently (within the last 14 days) undertaken shift work or rapid travel across time zones; (ii) declared health issues in the Physical Activity Readiness Questionnaire (PARQ); or (iii) had a mean chronotype score <22 or >43 based on the Composite Morningness questionnaire (CMQ) [28]. The mean chronotype score of the selected participants was intermediate (35.2 ± 4.5). Sleep Flexibility/Rigidity [F/R] score 43.4 ± 5.6 and Languid/Vigour [L/V] score 43.4 ± 9.3. All experimental tests were conducted in the United Kingdom between the months of March and May.

**Figure 1:**
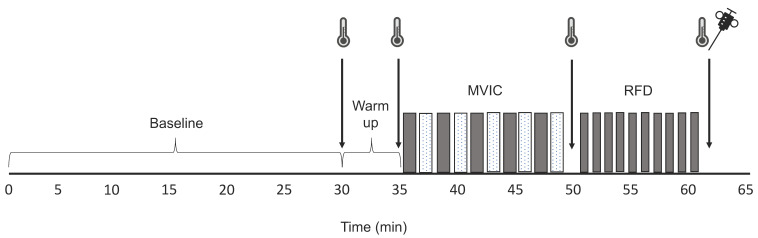
Experimental protocol. Schematic diagram of the experimental protocol undertaken by participants in the morning (07:30 h) and evening (17:30 h) interspersed by a 72-h wash-out period. Temperature data were collected from the rectum, muscle, and skin after a 30 min acclimatization period (baseline), after a cycle ergometry warm up (5 min at 150 W) and after periods of data collection, including the measurement of quadriceps maximal voluntary isometric contraction (MVIC) force and peak rate of force development (RFD). Participants performed 5 blocks of MVIC (dark bars) consisting of 1 unstimulated and 3 stimulated contractions, which were interspersed by rest periods (light bars). The peak RFD of the quadriceps muscle was assessed by 10 attempts (dark bars) interspersed by 30 s recovery periods. Samples of vastus lateralis muscle were taken by percutaneous needle biopsy immediately after RFD measurements.

Each participant completed 5 familiarization sessions (which occurred between 12:00 and 13:00 h), separated by 3-d intervals, to ensure full acquaintance with the experimental protocol. During each familiarization session, participants performed maximal voluntary isometric contractions (MVIC) of m. quadriceps femoris with electrical stimulation to assess the percentage activation (i.e., twitch interpolation technique) and trials designed to measure the peak rate of force development (RFD) of the same muscle. Thereafter, each participant completed a morning (07:30 h) and an evening (17:30 h) experimental session (Figure 1) separated by 3 d and assigned in a randomized counter-balanced order. Ten participants elected to undergo percutaneous needle biopsies, which were taken immediately after RFD measurements. Participants retired at 23:00 h and woke at 06:00 h to be in the laboratories for 07:00 h. All sessions took place under standard laboratory conditions (lighting, room temperature, and humidity = 200–250 lux, 19–20 °C, and 38–52%, respectively). Participants reported to the laboratory in a fasted (≥7 h) state for the morning session and were instructed not to consume food for ≥4 h prior to the evening session. Participants verbally stated they had complied with these restrictions on entry to the laboratory and recorded their dietary habits throughout the day including amount, type, and timing of food and fluid intake.

At the beginning of each experimental session, participants inserted a rectal probe approximately 10 cm past their external anal sphincter (Grant, Squirrel, Cambridge, UK). Skin thermistors (Grant, Cambridge, UK) were attached to four points of the body: sternum, upper arm (bicep mid-point), thigh (quadriceps mid-point), and calf (mid-point). Recordings of skin temperature (T_sk_) and rectal temperature (T_rec_) were taken every minute for 30 min (data used for analysis were averaged over the last 5 min), whilst participants remained awake in a semi-supine position. Muscle temperature (T_m_) was assessed in the vastus lateralis of the right leg using a needle thermistor (13050; ELAB, Rodovre, Denmark). T_rec_, T_sk_, and T_m_ were recorded after the active warm-up and after each MVIC and RFD attempt. Participants self-rated their perceived effort (0–10 Likert scale; “0” representing no effort and “10” maximal) after each MVIC and RFD exercise. 

Measurements of quadriceps femoris maximum voluntary isometric contraction (MVIC) force and peak RFD of the right leg were conducted using an isometric chair (Lido Active, Loredan, Davis, CA, USA). The position of the subject in the chair was standardized in accordance with the guidelines set by the manufacturer, and the hips and knee were secured at an angle of 90° with inextensible straps. MVIC was conducted with and without the interpolated twitch technique (ITT) to accustom participants to producing and maintaining MVIC force over the required (4 s) duration. For ITT contractions, the quadriceps femoris was electrically stimulated using two surface electrodes (7 × 12.7 cm Dura-stick II, Chattanooga Group, Hixson, TN, USA,), which were positioned on the anterolateral side of the thigh, on the belly of the rectus femoris and vastus lateralis muscles. An initial familiarization session was used to obtain maximal current (mA) tolerance and establish the supra-maximal current amplitude for superimposition during MVIC. Under resting conditions, the amperage of a 240 V square-wave pulse (100 μs, 1 Hz; Digimeter, DS7, Hertfordshire, UK) was progressively increased until further increases in intensity caused no further gain in twitch force [29]. Familiarization sessions were conducted until MVIC and RFD values reached a plateau, and the average voluntary muscle activation during the ITT was greater than 80%. N.B. ITT was used solely to determine the voluntary activation capacity and not to estimate the force of a maximally activated muscle. Force data were acquired (200 Hz) using a force transducer (KAP, Bienfait B.V. Haarlem, The Netherlands) and analyzed with commercially available software (AcqKnowledge III; Biopac Systems Inc, Aero Camino Goleta, CA, USA), which is consistent with previous work [10].

To measure quadriceps femoris peak RFD, participants performed maximum velocity contractions as forcefully as possible without countermovement. Visual data presentation was used to provide real-time ‘biofeedback’ and ensure no countermovement preceded each kick. Ten kicks were performed interspersed by 30 s rest periods (Figure 1). The peak RFD was defined as the highest positive value from the first derivative of the force signal (i.e., the greatest slope of the force–time curve). The 3 efforts of greatest force and no discernible counter-movement were selected for analysis [30].

A subset of 10 participants underwent a percutaneous needle biopsy of their right quadriceps (vastus lateralis) that was completed within 5 min of cessation of the muscle contraction protocol (Figure 1). Biopsies were performed under local anaesthesia (0.5% Marcaine) using a Bard Monopty 12-gauge × 10 cm length disposable core biopsy instrument (Bard Biopsy System, Tempe, AZ, USA). A 10 mm longitudinal stab incision was made, and the muscle fascia was pierced to enable the biopsy needle to be passed through the incision. Muscle samples (approximately 40 mg) were immediately frozen in liquid nitrogen and stored at −80 °C until further analysis. 

Muscle samples were ground under liquid nitrogen using a pestle and mortar. Muscle powders were accurately weighed and homogenized on ice in 10 volumes of 1% (*v*/*v*) Triton X-100, 40 mM Tris pH 7.4, including PhosSTOP^TM^ phosphatase inhibitor and cOmplete^TM^ protease inhibitor cocktail (Roche, Indianapolis, USA) using a glass–Teflon homogenizer. Homogenates were centrifuged at 1000× *g*, 4 °C for 5 min and supernatants containing soluble proteins were decanted and stored on ice. The pellet, which contains the majority of myofibrillar proteins, was resuspended in homogenization buffer and centrifuged at 1000× *g*, 4 °C for 5 min. The washed myofibrillar pellet was solubilized in Lysis buffer (7 M urea, 2 M thiourea, 4% (*w*/*v*) CHAPS, 30 mM Tris pH 8.5) and cleared by centrifugation at 12,000× *g*, 4 °C for 45 min. The protein concentrations of each myofibrillar and soluble protein fraction were measured using the Bradford assay (Sigma-Aldrich, Poole, Dorset, UK), and samples were normalized to 5 μg/μL in Lysis buffer. 

Top–down analysis of muscle protein modifications was performed by 2DGE as described previously [31]. Myofibrillar samples were resolved on immobilized pH gradient (IPG) strips (GE Healthcare, Little Chalfont, UK) by isoelectric focusing. The protein load was 250 μg and 50 μg per gel for broad-range (pH 3–11) and narrow-range (pH 4–7) gels, respectively. Second-dimension denaturing electrophoresis was performed through linear 11% poly-acrylamide gels. Proteins were stained with colloidal Coomassie blue (Bio-Rad Laboratories, Hercules, CA, USA). Digitized gel images (600 dpi, 16-bit grayscale) of samples taken from each participant in morning and evening conditions (n = 10 participants; total of 20 gels) were normalized by an inter-sample abundance ratio calculated from ‘non-changing’ features and between group differences were analyzed using SameSpots (TotalLab, Newcastle upon Tyne, UK), which is consistent with previous work [32].

Proteins were identified by reverse-phase liquid chromatography (LC)–electrospray ionization (ESI)–tandem mass spectrometry (MS/MS) of tryptic digests, as reported in [33]. Briefly, gel spots were excised from analytical gels (n = 20 biological replicates) or preparative gels of pooled samples and tryptic in-gel digestion was performed using an Xcise robot (Proteome Systems, North Ryde, Australia). LC-ESI-MS/MS was performed using a quadrupole-high capacity ion-trap mass spectrometer (HCT Ultra ETD II; Bruker Daltonics, Billerica, MA, USA) coupled via an ESI source to a nano-flow LC system (Ultimate 3000; Thermo Scientific, Waltham, MA, USA). Data-dependent MS/MS analysis in alternating collision-induced dissociation (CID) and electron-transfer dissociation (ETD) modes was performed, selecting the two most abundant precursor ions with active exclusion (30 s) enabled. Raw data were processed (Data Analysis 4.0, Bruker Daltonics, Billerica, MA, USA) and error tolerant searches of Mascot generic format files were performed against the UniProt database, which was restricted to Human using a locally implemented Mascot (www.matrixscience.com) server (version 2.2.03). The enzyme specificity was trypsin allowing 1 missed cleavage, carbamidomethyl modification of cysteine (fixed), oxidation of methionine (variable), and an *m/z* error of ± 0.5 Da. 

The tryptic digestion of soluble muscle proteins was performed using the filter-aided sample preparation (FASP) method. Aliquots containing 400 µg protein were washed with 200 µL of UA buffer (8 M urea, 100 mM Tris, pH 8.5). Proteins were incubated at 37 °C for 15 min in UA buffer containing 100 mM dithiothreitol followed by incubation (20 min at 4 °C) protected from light in UA buffer containing 50 mM iodoacetamide. UA buffer was exchanged for 50 mM ammonium bicarbonate, and sequencing-grade trypsin (Promega, Madison, WI, USA) was added at an enzyme to protein ratio of 1:50. Digestion was allowed to proceed at 37 °C overnight; then, peptides were collected in 100 μL 50 mM ammonium bicarbonate containing 0.2% trifluoroacetic acid. Peptide solutions were desalted using disposable Toptip C_18_ spin columns (Glygen Corp., Columbia, MD, USA) and vacuum centrifuged to dryness. Phosphopeptides were selectively enriched by binding to titanium dioxide (TiO_2_)-spin tips (Pierce), which was consistent with previous work [34]. Enriched phosphopeptide solutions were immediately adjusted to pH 4 with formic acid, vacuum centrifuged to dryness, and then resuspended in 0.1% formic acid (FA)prior to LC-ESI-MS/MS analysis.

The LC-ESI-MS/MS analysis of phosphopeptide-enriched samples was performed using an Ultimate 3000 RSLCTM nano system (Thermo Scientific, Waltham, MA, USA) coupled to a QExactive^TM^ mass spectrometer (Thermo Scientific, Waltham, MA, USA). Samples (5 μL each) were loaded by partial loop injection onto the trapping column (Thermo Scientific, PepMap100, C_18_, 75 μm × 20 mm) at a flow rate of 4 μL/min with 0.1% (*v*/*v*) trifluoroacetic acid (TFA) for 7 min. Samples were resolved on the analytical column (Easy-Spray C_18_ 75 μm × 500 mm 2 μm column) using a gradient of 97% A (0.1% FA) 3% B (99.9% ACN 0.1% FA) to 60% A 40% B over 90 min at a flow rate of 300 nL/min. The data-dependent program used for data acquisition consisted of a 70,000 resolution full-scan MS scan (AGC set to 10^6^ ions with a maximum fill time of 250 ms); the 10 most abundant peaks were selected for MS/MS using a 17,000 resolution scan (AGC set to 5 × 10^4^ ions with a maximum fill time of 250 ms) with an ion selection window of 3 *m/z* and a normalized collision energy of 30. To avoid the repeated selection of peptides, the MS/MS program used a 30 s dynamic exclusion window.

MS spectra were aligned using the Progenesis QI for Proteomics (Nonlinear Dynamics, Newcastle, UK). Prominent ion features were used as vectors to match each dataset to a common reference chromatogram. An analysis window of 9–99 min and 200–2000 *m/z* was selected encompassing charge states of ≤8. Peak lists were searched against the UniProt database and restricted to Human using a locally implemented Mascot (www.matrixscience.com) server (version 2.2.03). The enzyme specificity was trypsin, allowing one missed cleavage, fixed carbamidomethyl of cysteine, and variable oxidation of methionine and phosphorylation of serine (S), threonine (T), and tyrosine (Y). The instrument type was ESI-FTICR, and mass tolerances were 10 ppm for peptides and 0.01 Da for fragment ions. The Mascot output (xml format), which was restricted to non-homologous protein identifications (false discovery rate < 1%), was recombined with MS profile data in Progenesis QI for Proteomics, and peptide abundance data were normalized by the inter-sample abundance ratio calculated from ‘non-changing’ features. Peptide features with molecular weight search score (MOWSE) scores below the identity threshold (Mud-PIT scoring) were excluded. 

Data were analyzed in R version 3.5.2 and are presented as mean and standard deviation (SD) unless otherwise stated. Morning and evening MVIC force and peak RFD were compared with paired t-tests. Statistical analysis of proteomic data was conducted by within-subject one-way analysis of variance. Log transformed spot volumes or MS peptide data were normalized by inter-sample abundance ratio and used to investigate within-subject differences between morning and evening conditions. To determine the false discovery rate (FDR), p-value distributions were used to calculate q-values [35]. Functional annotation of proteomic data was conducted using the Search Tool for the Retrieval of Interacting Genes/Proteins [STRING; [36]. 

## 3. Results

### 3.1. Reliability and Diurnal Variation Data: MVIC, % Activation, RFD, and Subjects Rated Maximal Effort in n = 20 Subjects

**Figure 2:**
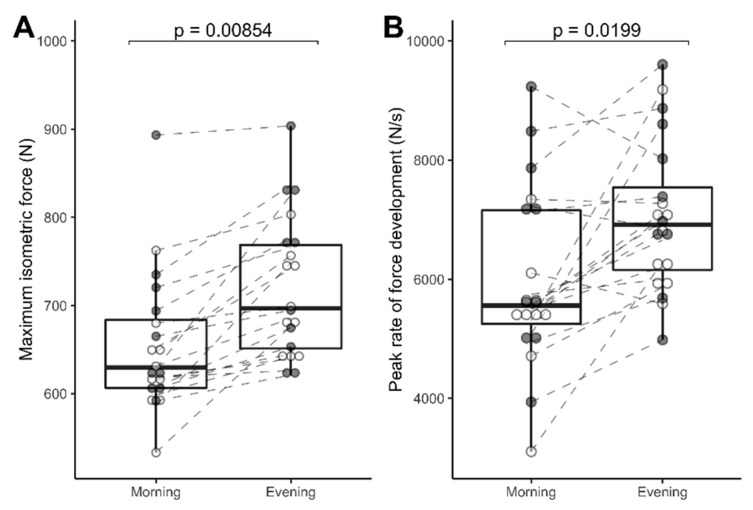
Diurnal differences in muscle force. Maximum voluntary isometric contraction (MVIC) force (**A**) and peak rate of force development; (**B**) of the quadriceps femoris muscle in the morning (08:00 h) and evening (18:00 h). Shaded (gray) data points represent data from n = 10 participants that gave muscle biopsy samples, whereas open (white) data points represent data from n = 10 participants that did not undergo biopsy procedures. Within-subject data are connected by dashed lines and group data (median, quartile ranges) are summarized by ‘box and whisker’ plots. Statistical differences were assessed by Student’s paired t-test, n = 20.

The intra-class correlation coefficient between MVIC force during the 4th and 5th familiarization sessions was 0.96 (95% CI, 0.91–0.99). MVIC force in the evening was 10.2% greater than in the morning (721 ± 80 versus 654 ± 79 N, *p* = 0.00854; Figure 2A). The intra-class correlation coefficient between peak RFD data collected during the 4th and 5th familiarization sessions was 0.45 (95% CI, 0.03–0.74). Peak RFD in the evening was 18% greater (7052 ± 1261 versus 5972 ± 1501 N/s, *p* = 0.0199; Figure 2B) than in the morning. The level of voluntary activation in the morning 82.8 ± 7.8% did not differ (*p* = 0.061) from the evening 86.5 ± 6.4%, and there were no significant difference in the rating of perceived effort. In all cases, participants rated 10/10 for effort regardless of whether the session was conducted in the morning or evening.

### 3.2. Data Handling: 10 Subjects from the Cohort of 20 Volunteered for Muscle Biopsy Procedures; Hence, Only Their Data (Temperature, MVIC, and RFD) Are Reported below to Align with the Proteomic Analyses

#### 3.2.1. Resting Rectal, Skin, and Muscle Temperatures (n = 10)

**Figure 3:**
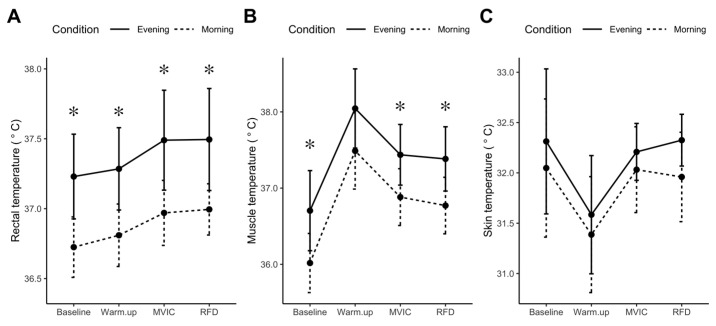
Diurnal differences in core, muscle, and skin temperature. Core (rectal) temperature (**A**), muscle temperature (**B**), and skin temperature (**C**), were recorded during the last 5 min of baseline and immediately after cycle ergometry warm-up, maximal voluntary isometric contraction (MVIC), or peak rate of force development (RFD) protocols. Data are presented as mean, standard deviation (n = 10), experimental condition is identified by morning (dashed line) and evening (solid line). * *p* < 0.05 statistical difference between morning and evening condition.

At rest, T_rec_ was greater in the evening (mean difference = 0.50 °C, *p* = 0.001, 95% CI: 0.27–0.74 °C) than morning (Figure 3A). T_m_ exhibited a similar diurnal variation (mean difference = 0.69 °C, *p* = 0.019, 95% CI: 0.14–1.23 °C; Figure 3B), whereas, T_sk_ exhibited no significant diurnal variation (mean difference = 0.26 °C, *p* = 0.377; Figure 3C). No significant interactions were found between temperature responses and time of day. The profiles of the temperature measurements (T_rec_, T_m_, and T_sk_) rose and fell in parallel over the three time points in both morning and evening conditions.

#### 3.2.2. MVIC, % Activation, Peak RFD, and Subject-Rated Maximal Effort (n = 10)

MVIC force in the evening was 9.2% greater (738 ± 98 versus 676 ± 92 N, *p* = 0.007) than in the morning (Figure 2A, closed/shaded data points). Peak RFD (7364 ± 1440 versus 6514 ± 1724 N/s, *p* = 0.064) was 13% greater in the evening (Figure 2B, closed/shaded data points). The level of voluntary activation for MVIC in the morning 79.6% did not differ (*p* = 0.056) from the evening 85.2%, and there were no significant differences in the rating of perceived effort. 

#### 3.2.3. Proteomic Data

**Figure 4:**
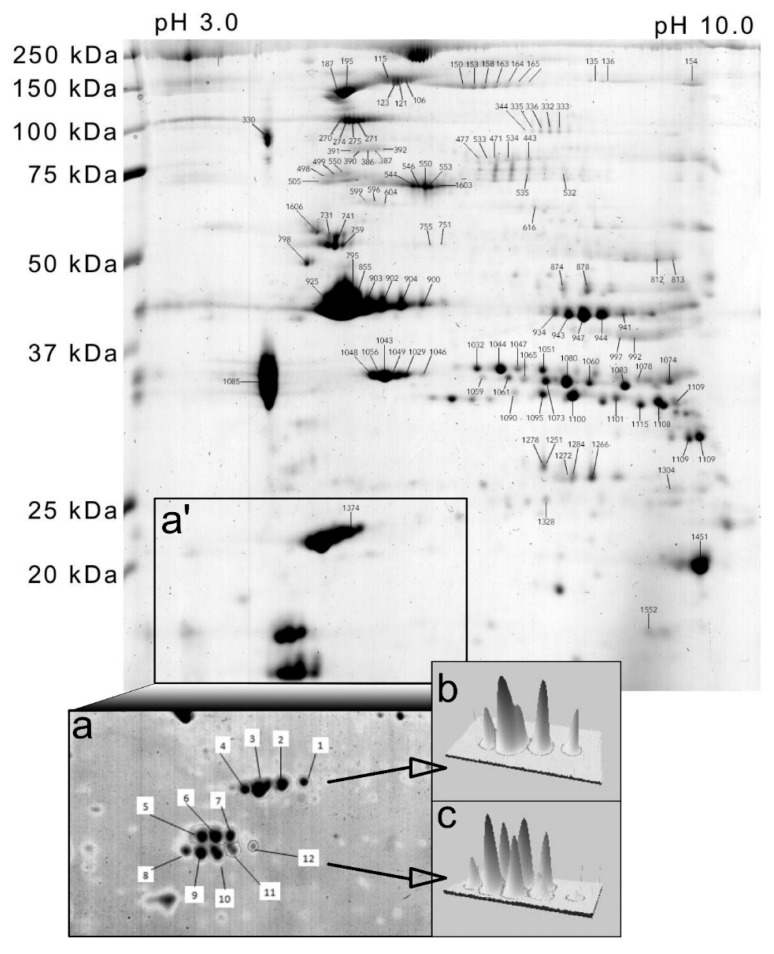
Analysis of myofibrillar proteoforms by two-dimensional gel electrophoresis. Gel map of human myofibrillar proteins annotated with spot numbers that correspond to protein identities in Table 1 and Table 2. Subpanels illustrate the region of interest (**a’**) and higher-resolution separation of region (**a’**) by narrow-range (pH 4–7) isoelectric focusing (**a**) to resolve myosin light chain isoforms. Panels (**b**,**c**) illustrate three-dimensional representations of essential myosin light chains (MyLC1/3) and regulatory myosin light chains MLRV and MLRS spots, respectively.

The analysis of human myofibrillar proteins by narrow range (pH 4–7) 2DGE (Figure 4 insets) was used to investigate differences in the relative abundance of proteoforms of essential and regulatory myosin light chains. Essential myosin light chains (MYLC1/3) were resolved as 4 proteoforms, slow-twitch/ventricular regulatory myosin light chains (MLRV) were resolved as 3 proteoforms, and fast-twitch/skeletal muscle myosin regulatory light chains (MLRS) were resolved as 4 proteoforms. There were no statistically significant differences in myosin light chain proteoform abundance between morning and evening conditions (Table 1).

A broader range (pH 3–11) of 2DGE resolved 122 protein spots (Figure 4 and Table 2). In total, 40 non-redundant proteins were identified, i.e., the majority of proteins were detected in multiple gel spots that represent different proteoforms. The abundance of 6 proteoforms was statistically (*p* < 0.05) different between morning and evening conditions (Table 2). In the evening, greater abundances were observed in myosin binding protein C1 (80%), glycogen phosphorylase (43%), and beta enolase (24%), whereas the abundances of myosin binding protein H (−18%), nebulin (−48%), and troponin T slow (−22%) were less in the evening compared to the morning. The greatest difference was in the abundance of the slow isoform of myosin binding protein C (MYBPC1), which was resolved as a series of 6 proteoforms. Therefore, the difference in abundance of spot ^#^ 164 reported here may represent a change in post-translational modification. LC-ESI-MS/MS analysis of all MYBPC1 proteoforms (spot numbers: 150, 153, 158, 163, 164, and 165) did not unambiguously identify site-specific modifications that were unique to a particular MYBPC1 proteoform. The phosphorylation of MYBPC1 S^178^ was detected in two MYBPC1 proteoforms (spots 153 and 158), and therefore, it was not specifically related to time-of-day differences in the pattern of MYBPC1 proteoforms.

**Figure 5:**
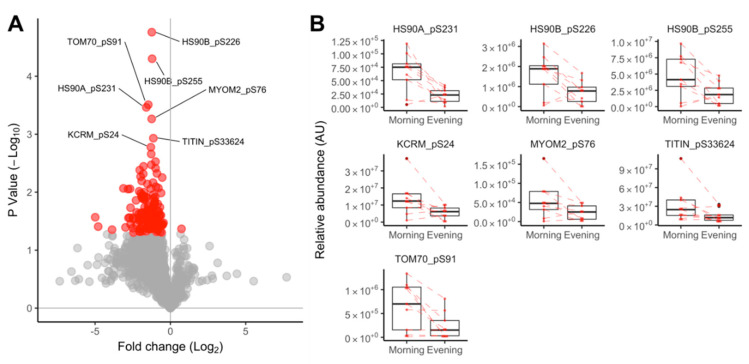
Diurnal differences in muscle protein phosphorylation. Volcano plot (**A**) presenting the log_2_ fold change in phosphopeptide abundance between evening and morning conditions and the statistical significance determined by within-subject ANOVA (n = 9 participants). Phosphopeptides that were statistically different (*p* < 0.05) and had a false discovery rate <10% are annotated with their unique UniProt identifier and phosphorylation site; phosphopeptide-specific data (**B**) illustrating within-subject data connected by dashed lines and group data (median, quartile ranges) summarized by ‘box and whisker’ plots for the statistically significant (*p* < 0.05, FDR < 10%) phosphopeptides.

**Figure 6:**
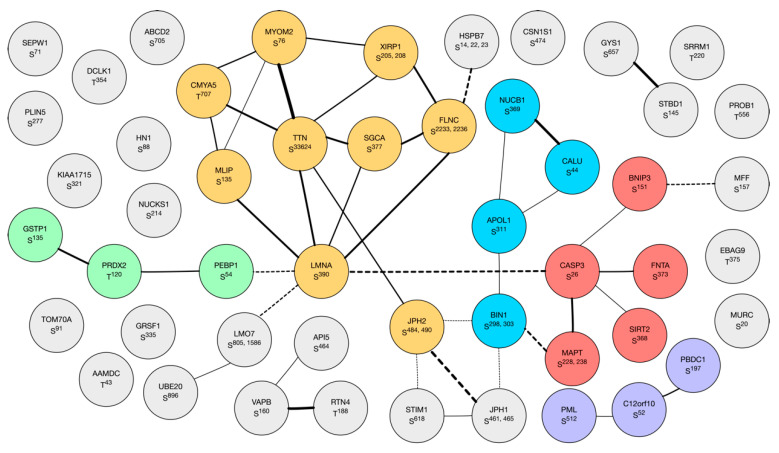
Network of phosphorylated proteins. Network of known and predicted interactions between proteins (n = 141) with phosphopeptides that exhibited statistically significant (*p* < 0.05) differences in phosphorylation between morning and evening conditions. The network was constructed using the Search Tool for the Retrieval of Interacting Genes/Proteins (STRING), and subsets of proteins were highlighted by k-means clustering (6 groups). Node annotations represent gene names and phosphorylation sites. The width of the vectors indicate levels of evidence for protein interconnections (e.g., interaction, co-expression, or bibliometric data). Group 1 (orange) contains proteins associated with the myofibrillar apparatus and muscle cytoskeleton. Group 2 (red) is characterized by proteins involved in apoptosis (e.g., caspase 3, CASP3). Group 1 and 2 are interconnected by myc box-dependent-interacting protein 1 (BIN1; blue) which may be a key component of the muscle M-Band in addition to its role associated with t-tubules. Smaller groups are characterized by calcium-handling proteins (purple) or redox enzymes (green).

The label-free quantitation of TiO_2_-enriched human muscle peptides profiled 1693 phosphopeptides (721 proteins) in n = 9 paired samples (1 sample was lost during processing) taken in the morning and evening condition. Generally, the abundance of muscle phosphopeptides was greater in the morning than evening (Figure 5A). One-hundred and forty phosphopeptides from 104 proteins were statistically different (*p* < 0.05) at a false discovery rate (FDR) of 22% (Figure 5A), and 7 peptides had a false discovery rate of <10% (Figure 5B). Of 141 peptides at *p* < 0.05, 131 are reported in PhosphositePlus^®^ (https://www.phosphosite.org), 122 have high throughput (HTP) associated papers with PubMed identifiers (PMID), 46 had low throughput (LHP) PMID associated papers, and 10 were new or have not been reported previously. Phosphopeptide abundance data including literature links are available online at 10.6084/m9.figshare.10316624 (Supplementary Material). Network analysis of 104 significant proteins using STRING (Figure 6) highlighted clusters including (i) myofibrillar proteins centering around titin and myomesin; (ii) apoptosis-related proteins centering around caspase; and (iii) redox proteins including glutathione transferase and peroxiredoxin 2. 

## 4. Discussion

We report widespread differences in the post-translational state of human muscle proteins after exercise in the morning (08:00 h) and evening (18:00 h). In particular, our data indicate differences in the phosphorylation of proteins within or close to the muscle M-band that could relate to the well-established morning versus evening differences in muscle isometric force. Our novel analysis also adds to emerging evidence regarding the daily timing of exercise and the use of exercise as a Zeitgeber for optimal muscle function [37]. The current investigation was limited to the interaction between exercise and time of day; therefore, further work is required to investigate the main effects of exercise or time of day in isolation. For example, it will be important to resolve which of the differences in protein post-translational modification are intrinsic diurnal differences and occur in the absence of the exercise stimulus.

**Figure 7:**
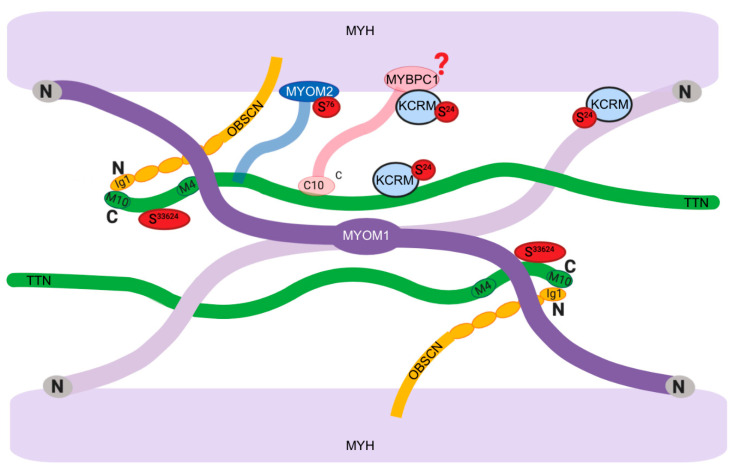
Diurnal differences in M-band phosphorylation. Diagrammatic representation of M-band protein interactions redrawn from [38]. Myomesin 1 (MYOM1) and obscurin (OBSCN) are principal M-band proteins [39]. The N-terminal regions of MYOM1 interact with myosin heavy chain (MYH), titin (TTN), and muscle creatine kinase (KCRM), whereas the C-terminal regions of MYOM1 dimerize in an anti-parallel arrangement that corresponds with the M-bridges [40]. MYOM1 interacts with the C-terminal regions of TTN within the M-band. The TTN kinase domain (resides 32,178–32,432) includes the regulatory Y^32341^ residue [41], whereas we discovered that the phosphorylation of S^33624^ of TTN is greater in the morning than evening. Myomesin 2 (MYOM2, formally known as M-Protein) interacts with the light meromyosin (LMM) region of MYH and the C-terminal region of TTN [42]. The phosphorylation of MYOM2 S^76^ disrupts the binding of MYOM2, and LMM [43] and is more prevalent after exercise in the morning compared to evening. Myosin binding protein C (MYBPC1) localizes to the M-band through its interaction with obscurin [44]. MYBPC1 is required for the binding of muscle creatine kinase (KCRM) to the M-band region. MYBC1 exhibits differences in post-translational state between morning and evening, which are likely to be phosphorylation [45]. KCRM S^24^ phosphorylation is greater in the morning than evening. The N-terminal region of KCRM is necessary for the creatine/phosphocreatine- and pH-dependent interaction of KCRM with the M-band [46].

The unsupervised network analysis of proteins that contained significant time-of-day differences in phosphorylation status (Figure 6) highlighted a cluster of myofibrillar proteins, including prominent links between titin (TTN) and myomesin 2 (MYOM2). TTN and MYOM2 were also amongst the most highly significant (*p* < 0.05, FDR < 10%) differences (Figure 5B) alongside muscle creatine kinase (KCRM), heat shock 90 proteins (HS90A and HS90B), and the mitochondrial importer receptor subunit (TOM70). With the exception of TOM70, a commonality shared amongst these proteins is their involvement in, or localization to, the sarcomeric M-band (Figure 7).

The M-band is a key component of the sarcomeric structure that distributes force across opposing myosin thick filaments [38] and is well-placed to mediate mechanical signals to the nucleus and wider muscle proteome. MYOM2, formerly known as 165 kDa M-protein, is a major component of the M-Band and is specifically expressed in cardiac and fast-twitch skeletal muscle [47]. MYOM2 binds to the light meromyosin (LMM) tail of myosin heavy chains in the M-band region and the phosphorylation of MYOM2 S^76^ by protein kinase A (PKA) disrupts the binding of MYOM2 and LMM in vitro [43]. We report a significantly greater phosphorylation of MYOM2 S^76^ in the morning, which could indicate alteration to the M-band structure and the disruption of force transmission in vivo. The circadian regulation of this effect is supported by data from animals carrying clock gene mutations that exhibit a decrement in myofiber-specific force and disruption to the hexagonal structure of thick filament proteins [17]. In the future it will be of interest to investigate whether differences in protein phosphorylation also exist between the muscle of wild-type and clock mutant mice.

We report that the phosphorylation of TTN S^33624^ near the C-terminus of titin is greater in the morning than evening in human muscle. The kinase domain of human titin includes Y^32341^ [41]. The TTN S^33624^ site reported here has been previously detected in phosphoproteome maps of human skeletal muscle [26], but as of yet, no function is assigned to S^33624^ phosphorylation. Titin is involved in sensing mechanical strain via its C-terminal kinase domain located at the sarcomeric M-band [48]. Mechanical strain causes unfolding of the kinase domain, which occurs prior to unfolding of the structural titin Ig/Fn domains. The kinase domain of titin is involved in gene expression of muscle E3 ligases in response to mechanical strain [49]. If TTN S^33624^ phosphorylation alters the stretch responsiveness of titin kinase, this could raise questions regarding potential responsiveness to training in morning versus evening.

Contrary to our expectation, in-depth analysis of myosin light chain proteoforms found no differences between morning and evening conditions (Table 1). In skeletal muscle, the phosphorylation of myosin regulatory light chain (MLRS) contributes to twitch potentiation in fast-twitch fibers [20]. However, we report that the post-translational modification of neither essential nor regulatory myosin light chain isoforms is associated with diurnal differences in maximum isometric force in human muscle. Further analysis of myofibrillar proteoforms using broader range (pH 3–10) isoelectric focusing (Figure 4) discovered putative post-translational modifications of myofibrillar proteins and metabolic enzymes in human muscle (Table 2) that raise new hypotheses regarding the differences in muscle force output. MYBPC1 is of interest because it is a major component of the muscle thick filament [50]. Proteomic analysis of hearts from wild-type and cardiac clock mutant mice [45] reported circadian differences in the level of phosphorylation of MYBPC1 using the phosphor-specific, Pro-Q diamond, stain. The phosphorylation of MYBPC1 is associated with enhanced contractility in response to beta-adrenergic receptor stimulation [51], and MYBPC1 can be phosphorylated at numerous sites by protein kinase A (PKA) and protein kinase C [52]. We were unable to unambiguously identify the site-specific modification of MYBPC1 that differed in human muscle between the morning and evening conditions. Nevertheless, the consistency in findings between the heart [45] and human skeletal (current work) muscle indicate that further targeted analysis on MYBPC1 proteoforms is warranted.

Muscle creatine kinase (KCRM, previously known as MM-CK) is a prominent M-band protein and key enzyme in high-energy phosphate metabolism [53]. MYBPC1 is necessary for the recruitment of KCRM to myosin, and this interaction is dependent on both the ratio of creatine/phosphocreatine and intracellular pH [53]. The interaction between KCRM and the M-band weakens as the intracellular pH rises and the ratio of creatine/phosphocreatine increases [53]. KCRM also interacts with immunoglobulin-like domains near the C-terminal of myomesin and M-protein (MYOM2; [46]). The site (S^24^) of KCRM phosphorylation reported here (Figure 5) is immediately adjacent to K^25^, which is one of 4 lysine residues near the N-terminus of KCRM that are required for the pH-dependent interaction between KCRM and M-band proteins [46]. The phosphorylation of KCRM S^24^ has been previously detected in phosphoproteome maps of human muscle [26], but the upstream kinase and functional consequences of KCRM S^24^ phosphorylation remain to be elucidated. Based on our current findings, it may be hypothesized that KCRM S^24^ phosphorylation has a disruptive influence and may weaken the interaction between KCRM and the M-band region.

In addition to sarcomeric proteins, isoforms of heat shock protein 90 (HSP90) and the mitochondrial import receptor protein (TOM70) also exhibited a diurnal difference in phosphorylation status. HSP90 does not localize to the M-band, but it is required for the chaperone-mediated assembly of myosin in muscle [54]. HSP90 modulates the myosin replacement rate into myofilaments and influences the expression of myosin genes [55]. Post-translational modifications, including the phosphorylation, of HSP90 are recognized to modulate its chaperone activity [56], but an in-depth understanding of the effects of different site-specific modifications is not yet available. The phosphorylation of S^226^ and S^255^ of HS90B and S^231^ HS90A reported here (Figure 5) is mediated by casein kinase 2 (CK2), which is known to modulate the molecular clock through the phosphorylation of Period 2 S^53^ [57]. TOM70 S^91^ is reported to exhibit a circadian rhythm in mouse liver [58], and the deletion of BMAL1 causes muscle mitochondrial dysfunction [17]. The phosphorylation of TOM70 S^174^ by PKA inhibits its receptor activity [59], but the effect of S^91^ phosphorylation, reported here, remains to be determined. The future phosphoproteomic analysis of mitochondria may give further insight to time-of-day differences in the response of muscle metabolism to exercise [16].

Findings from our non-targeted analysis of protein phosphorylation suggest a connection to PKA. Several of the phosphorylation sites on myofibrillar proteins have been reported to be targets of PKA, and we detected greater phosphorylation of the catalytic subunit of PKA on threonine (T)^198^ in morning samples. PKA is a key second messenger of muscle beta-adrenergic receptor signaling, and adrenal hormones, including catecholamines and glucocorticoids, have a role in synchronizing circadian rhythms across peripheral tissues in vivo [60]. Glucocorticoids are regarded as the main synchronizing hormones [61], but the circadian control of liver metabolism in mice [62] has been linked with adrenergic receptor signaling. The acrophase (approximately mid-day) in plasma catecholamines in humans [63] does not coincide with the morning–evening differences in muscle isometric force. However, plasma catecholamine response to exercise is greater in the morning than evening [63]. There is a well-established interaction between glucocorticoids and adrenergic receptor signaling [64], and the early morning peak in cortisol could potentiate responses to adrenaline (consistent with our data). Glucocorticoid stimulation may enhance the intracellular 3’,5’-cyclic adenosine monophosphate (cAMP) response to beta-adrenergic receptor stimulation, but this might be expected to result in greater muscle force output [65] in the morning. The acrophase of plasma cortisol early in the morning coincides with the bathyphase of muscle force production. The phosphorylation of PKA T^198^ is mediated by phosphatidylinositol 3-kinase [66], which opens the possibility of upstream regulation by interaction between the glucocorticoid receptor and p85α subunit of phosphatidylinositol 3-kinase [67]. Our data provide the basis for investigating the role of glucocorticoids in the regulation of cAMP in the context of time-of-day differences in muscle force.

The primary aim of this study was to investigate whether modifications occur to the contractile proteins of skeletal muscle that associate with its ability to generate force at the two different times of day. Biopsies were performed to investigate whether modifications co-occur to the contractile proteins of skeletal muscle that affect its ability to produce force at each time of day. A limitation of our work is that baseline samples were not collected prior to exercise, so it remains to be shown whether the time-of-day differences in protein post-translational modification exist in muscle that has not performed exercise. Overall, the phosphorylation of muscle proteins was greater after exercise in the morning than in the evening, which may indicate a diurnal difference in muscle protein phosphatase activity. The modulation of protein phosphatase 1 activity alters circadian rhythms in flies [68], but the selective dephosphorylation of proteins in human muscle remains to be investigated. We principally studied phosphorylation, but other post-translational modifications may also be important. The majority of covalent protein modifications change the isoelectric point of proteins, which makes 2DGE an elegant tool for the non-targeted discovery of protein modifications. However, the relatively small samples extracted from 2DGE spots, particularly in human biopsy samples where the starting material is also limited, make this work challenging. A third limitation concerning our study was the selection of participants for biopsies; in our case, we biopsied only those who were happy to give consent. Even though significant diurnal differences were found in expected parameters, future work should establish diurnal variation first within the population who have consented to have biopsies and select those participants who have the greatest variation. 

## 5. Conclusions

Exercise in the morning, compared to the evening, resulted in the greater phosphorylation of proteins in human muscle that are components of or are associated with the M-band region. The phosphorylation of these proteins may alter the M-band structure and disrupt force transmission, thus potentially explaining the lower force output in the morning compared to evening. Our findings in humans add to data from animal models where deletion of the clock protein, BMAL1, results in disorganization of the 3D myofibrillar lattice and a loss in muscle force. The data generated in human muscle provide new targets to investigate the modulation of muscle force output or the application of exercise as a Zeitgeber of muscle function.

## Figures and Tables

**Table 1:** Myosin light chain proteoform abundances.

UniProt ID	Description	Spot ^#^	Morning	Evening	*p*
MYL1/3	Essential myosin light chain 1/3	1	924 (498)	785 (612)	0.727
		2	4426 (1504)	3968 (1984)	0.884
		3	10,942 (3173)	9788 (4894)	0.485
		4	2378 (808)	2192 (1293)	0.431
MLRV	Regulatory myosin light chain, slow/ventricular	5	2675 (1524)	2985 (2179)	0.498
		6	4176 (1837)	3699 (2182)	0.459
		7	1940 (950)	1600 (10,089)	0.389
MLRS	Regulatory myosin light chain, fast/skeletal	8	1557 (1027)	2206 (1875)	0.216
		9	4675 (1589)	5214 (3128)	0.151
		10	4420 (1149)	3762 (1768)	0.467
		11	1286 (540)	982 (569)	0.426
HSPB6	Heat shock protein 20	12	282 (214)	0.00 (0.00)	-

Spot ^#^ correspond with the 2D gel map (Figure 5 inset a). UniProt identifier and protein description based on peptide mass spectrometry of in-gel protein digests. Normalized spot abundances (AU) in the morning and evening are presented as mean (SD) and statistical difference (*p*) was determined by within-subject ANOVA (n = 10).

**Table 2:** Diurnal differences in myofibrillar proteoform abundance.

Spot ^#^	UniProt ID	Protein Name	MOWSE	% Seq	Anova (p)	FC	Morning Mean	Morning SD	Evening Mean	Evening SD
335	PYGM	Glycogen phosphorylase, muscle form	1938	53	0.0155	1.43	245	44	350	104
163	MYPC1	Myosin-binding protein C, slow-type	2135	42	0.0226	1.80	343	86	617	357
703	MYBPH	Myosin-binding protein H	425	23	0.0313	1.17	133	28	114	26
874	ENOB	Beta-enolase	1040	55	0.0335	1.24	814	199	1007	174
1046	TNNT1	Troponin T, slow skeletal muscle	738	32	0.0433	1.22	239	71	196	45
154	NEBU	Nebulin	964	6	0.0435	1.48	959	352	648	252
391	MYH2	Myosin-2	1510	18	0.0546	1.37	239	56	329	158
881	KCRM	Creatine kinase M-type	922	54	0.0570	1.17	535	74	625	118
795	ACTS	Actin, alpha skeletal muscle	1011	54	0.0620	1.47	271	114	185	56
742	ACTN2	Alpha-actinin-2	80	4	0.0727	1.25	510	102	408	134
1059	TNNT3	Troponin T, fast skeletal muscle	394	24	0.0735	1.32	244	84	186	54
992	ALDOA	Fructose-bisphosphate aldolase A	733	58	0.0929	1.19	472	126	559	138
903	ACTS	Actin, alpha skeletal muscle	1009	62	0.0931	1.67	16,872	10,694	10,109	2314
135	MYPC2	Myosin-binding protein C, fast-type	2015	45	0.0978	1.23	168	54	137	41
1357	KCRM	Creatine kinase M-type	466	32	0.0978	1.43	2542	1135	1782	577
686	NEBU	Nebulin	2181	13	0.1017	1.17	336	66	394	69
386	MYH2	Myosin-2	1266	13	0.1048	1.27	233	78	295	104
739	DESM	Desmin	1884	63	0.1072	1.20	1176	181	979	292
1043	TNNT1	Troponin T, slow skeletal muscle	599	33	0.1143	1.28	884	293	689	278
596	MYH7	Myosin-7	1229	17	0.1321	1.15	259	66	298	49
392	MYH2	Myosin-2	709	12	0.1328	1.17	109	44	128	36
390	MYH2	Myosin-2	1367	14	0.1481	1.28	177	40	227	89
855	ACTS	Actin, alpha skeletal muscle	1319	65	0.1531	1.25	18,032	6216	14,461	4631
158	MYPC1	Myosin-binding protein C, slow-type	2460	49	0.1606	1.32	547	224	721	297
387	MYH2	Myosin-2	915	12	0.1636	1.24	173	85	214	71
685	KPYM	Pyruvate kinase isozymes M1/M2	1329	51	0.1662	1.17	398	68	466	112
731	DESM	Desmin	2161	72	0.1818	1.16	2919	408	2527	728
153	MYPC1	Myosin-binding protein C, slow-type	2648	51	0.1857	1.34	518	218	694	314
344	PYGM	Glycogen phosphorylase, muscle form	1560	43	0.1876	1.53	198	48	304	242
741	DESM	Desmin	1927	67	0.1927	1.17	3290	778	2811	997
544	ALBU	Serum albumin	1539	56	0.2047	1.25	682	203	852	348
389	TNNT1	Troponin T, slow skeletal muscle	153	23	0.2070	1.16	558	103	645	155
1065	TNNT3	Troponin T, fast skeletal muscle	421	26	0.2103	1.18	542	124	460	117
164	MYPC1	Myosin-binding protein C, slow-type	2581	51	0.2105	1.25	337	57	423	190
751	TBA4A	Tubulin alpha-4A	467	29	0.2232	1.14	312	60	274	43
997	ALDOA	Fructose-bisphosphate aldolase A	812	60	0.2234	1.21	652	188	789	289
546	ALBU	Serum albumin	1622	59	0.2244	1.34	1381	468	1850	766
812	ATPA	ATP synthase subunit alpha, mitochondrial	1275	45	0.2274	1.15	688	164	790	219
150	MYPC1	Myosin-binding protein C, slow-type	2849	50	0.2300	1.30	442	236	575	280
813	ATPA	ATP synthase subunit alpha, mitochondrial	963	42	0.2328	1.10	755	167	829	176
1606	VIME	Vimentin	1861	69	0.2342	1.35	707	339	955	389
1105	TPM2	Tropomyosin beta chain	567	32	0.2501	1.19	454	120	381	80
1251	CAH3	Carbonic anhydrase 3	419	44	0.2622	1.14	829	240	944	230
878	ENOB	Beta-enolase	1196	53	0.2624	1.19	1109	283	1325	358
616	CATA	Catalase	710	38	0.2974	1.77	832	103	1476	1963
941	KCRM	Creatine kinase M-type	890	54	0.3013	1.14	1295	299	1480	439
947	KCRM	Creatine kinase M-type	1295	57	0.3075	1.14	6801	1461	7786	1689
1057	ACTC	Actin, alpha cardiac muscle 1	52	11	0.3596	1.16	329	118	383	185
505	HSP7C	Heat shock cognate 71 kDa protein	1246	39	0.3731	1.19	1386	475	1643	850
500	HSP71	Heat shock 70 kDa protein 1A/1B	1470	46	0.3816	1.02	583	99	570	136
1085	TPM3	Tropomyosin alpha-3 chain	1478	72	0.3846	1.10	37,171	11,538	33,691	9083
1603	ALBU	Serum albumin	1625	58	0.3869	1.22	714	404	867	335
1097	TNNT1	Troponin T, slow skeletal muscle	576	32	0.3914	1.20	1448	656	1207	609
553	ALBU	Serum albumin	1838	60	0.3938	1.29	4881	2765	6293	3338
902	ACTS	Actin, alpha skeletal muscle	962	63	0.4326	1.25	8992	5539	7196	2076
1374	MYL3	Myosin light chain 3	856	77	0.4537	1.10	8098	3473	7382	2866
1078	MYOZ1	Myozenin-1	848	85	0.4556	1.00	401	76	402	239
1336	TPIS	Triosephosphate isomerase	929	76	0.4585	1.04	316	48	304	100
1103	KCRM	Creatine kinase M-type	418	29	0.4598	1.40	623	607	446	123
944	KCRM	Creatine kinase M-type	1100	53	0.4644	1.12	3853	690	4310	1195
900	ACTS	Actin, alpha skeletal muscle	415	39	0.4649	1.21	3869	2169	3198	713
1272	MYOZ1	Myozenin-1	154	29	0.4785	1.05	355	88	373	47
532	PGM1	Phosphoglucomutase-1	876	37	0.4889	1.09	446	125	409	80
106	MYH2	Myosin-2	3726	33	0.4924	1.59	550	340	874	888
1090	TNNT3	Troponin T, fast skeletal muscle	520	32	0.5034	1.12	591	178	528	89
333	PYGM	Glycogen phosphorylase, muscle form	1611	47	0.5039	1.21	164	33	198	95
1503	KCRM	Creatine kinase M-type	151	13	0.5085	1.06	1063	345	1007	590
1032	TNNT3	Troponin T, fast skeletal muscle	491	27	0.5192	1.23	1032	534	837	258
943	KCRM	Creatine kinase M-type	1126	59	0.5267	1.06	2690	574	2846	551
1056	TNNT1	Troponin T, slow skeletal muscle	687	37	0.5340	1.13	8515	4319	7506	4221
1049	TNNT1	Troponin T, slow skeletal muscle	652	36	0.5550	1.10	1792	601	1630	818
165	MYPC1	Myosin-binding protein C, slow-type	2366	51	0.5618	1.14	323	69	368	161
1266	TPIS	Triosephosphate isomerase	932	65	0.5624	1.07	1389	280	1489	354
1132	MYH2	Myosin-2	616	8	0.5679	1.05	1154	324	1209	255
604	MYH2	Myosin-2	1104	13	0.5805	1.05	199	43	189	41
121	MYH2	Myosin-2	4044	37	0.5910	1.51	691	599	1041	1196
455	KPYM	Pyruvate kinase isozymes M1/M2	815	36	0.6060	1.06	856	158	810	117
550	ALBU	Serum albumin	1852	65	0.6109	1.23	2514	1360	3093	1897
1074	G3P	Glyceraldehyde-3-phosphate dehydrogenase	1068	54	0.6170	1.03	3635	771	3532	1105
925	ACTS	Actin, alpha skeletal muscle	1277	63	0.6537	1.05	73,798	17,469	77,544	17,257
123	MYH2	Myosin-2	3325	34	0.6580	1.31	819	851	1072	1290
798	ATPB	ATP synthase subunit beta, mitochondrial	1882	68	0.6678	1.13	947	356	1069	547
1083	TNNT3	Troponin T, fast skeletal muscle	583	34	0.6867	1.12	2406	775	2689	1066
136	MYPC2	Myosin-binding protein C, fast-type	1838	41	0.6868	1.07	223	72	207	45
1073	TNNT3	Troponin T, fast skeletal muscle	482	26	0.6887	1.09	1620	579	1492	603
1048	TNNT1	Troponin T, slow skeletal muscle	738	32	0.6912	1.25	3355	2869	2681	1052
471	MYH2	Myosin-2	1204	15	0.6940	1.09	202	91	186	98
934	KCRM	Creatine kinase M-type	1134	54	0.6981	1.03	1253	233	1222	210
1115	MYOZ1	Myozenin-1	378	30	0.7011	1.05	1462	416	1393	415
904	ACTS	Actin, alpha skeletal muscle	877	55	0.7143	1.01	9431	1361	9383	3034
443	MYH2	Myosin-2	978	13	0.7267	1.05	331	103	348	96
1381	KCRM	Creatine kinase M-type	469	28	0.7267	1.08	24,048	9478	22,334	6612
115	MYH7	Myosin-7	4288	35	0.7551	1.14	2646	2960	3028	3141
1061	TNNT3	Troponin T, fast skeletal muscle	457	36	0.7595	1.28	540	382	424	155
1044	ACTC	Actin, alpha cardiac muscle 1	342	28	0.7668	1.14	2912	1480	2552	917
1080	ACTC	Actin, alpha cardiac muscle 1	183	23	0.7707	1.06	4741	1932	4479	2310
270	ACTN2	Alpha-actinin-2	3558	69	0.7779	1.08	2145	422	2326	812
271	ACTN2	Alpha-actinin-2	3354	71	0.7866	1.04	679	243	706	256
274	ACTN2	Alpha-actinin-2	3445	69	0.7899	1.07	1058	239	1128	390
187	MYH7	Myosin-7	3534	31	0.7939	1.55	6318	8061	4082	3064
1051	TNNT3	Troponin T, fast skeletal muscle	507	36	0.8007	1.11	1454	657	1312	446
1108	MYOZ1	Myozenin-1	708	50	0.8010	1.02	3435	1569	3513	1097
332	PYGM	Glycogen phosphorylase, muscle form	1651	44	0.8203	1.01	295	50	299	93
330	TPM1	Tropomyosin alpha-1 chain	947	47	0.8216	1.07	1964	874	1834	716
1100	TNNT3	Troponin T, fast skeletal muscle	474	37	0.8243	1.12	4641	2707	4144	1391
1095	TNNT3	Troponin T, fast skeletal muscle	452	28	0.8373	1.09	1477	719	1355	374
499	HSP71	Heat shock 70 kDa protein 1A/1B	1070	39	0.8419	1.02	516	114	508	118
980	TNNT1	Troponin T, slow skeletal muscle	696	35	0.8427	1.01	1131	271	1146	265
1198	LDB3	LIM domain-binding protein 3	609	17	0.8445	1.01	2437	827	2415	1038
899	DESM	Desmin	198	11	0.8495	1.08	1329	574	1230	235
1129	VDAC1	Voltage-dependent anion-selective channel protein 1	612	59	0.8774	1.10	383	206	347	82
1552	HERC1	Probable E3 ubiquitin-protein ligase HERC1	52	2	0.8852	1.06	1557	547	1652	1207
1101	TNNT3	Troponin T, fast skeletal muscle	540	33	0.9155	1.01	1068	440	1077	383
477	MYH2	Myosin-2	2036	26	0.9173	1.08	564	398	609	497
533	MYH2	Myosin-2	1084	13	0.9250	1.27	365	409	289	165
1397	ENOB	Beta-enolase	266	26	0.9287	1.02	12,335	3415	12,540	3761
535	MYH2	Myosin-2	1195	17	0.9394	1.23	456	482	370	163
1451	TNNI2	Troponin I, fast skeletal muscle	378	50	0.9545	1.02	229	127	225	130
479	MYH2	Myosin-2	1359	18	0.9612	1.04	543	385	563	481
275	ACTN2	Alpha-actinin-2	3512	71	0.9648	1.04	925	221	965	355
534	MYH2	Myosin-2	1360	16	0.9683	1.14	588	587	515	327
336	PYGM	Glycogen phosphorylase, muscle form	1778	50	0.9720	1.02	310	96	305	69
1251	HBB	Hemoglobin subunit beta	441	60	0.9780	1.00	834	137	837	171
195	MYH7	Myosin-7	4307	32	0.9894	1.34	2446	3633	1821	1188
1060	TNNT3	Troponin T, fast skeletal muscle	481	26	0.9915	1.01	2012	749	2001	801
1034	TPM2	Tropomyosin beta chain	1450	69	0.9963	1.01	22,483	6641	22,339	5236

Spot ^#^ correspond with the 2D gel map (Figure 4). UniProt identifier and protein name based on peptide mass spectrometry of in-gel protein digests searched against human entries in the SwissProt database using Mascot. The probability-based molecular weight search score (MOWSE) and percentage of protein sequence identified are presented. Spot abundances (AU) in the morning and evening are presented as mean (SD) and statistical difference (P) was determined by within-subject ANOVA (n = 10). Fold change (FC) is spot abundance in the evening compared to the morning condition.

## Supplementary Materials

Phosphopeptide data: https://www.mdpi.com/2227-7382/8/3/22/s1.
